# Care coproduction strategies based on the experience of patients hospitalized for COVID-19: a multicenter qualitative study*

**DOI:** 10.1590/1980-220X-REEUSP-2025-0354en

**Published:** 2026-09-18

**Authors:** Jhonata de Souza Joaquim, Diovane Ghignatti da Costa, Alacoque Lorenzini Erdmann, Franciely Daiana Engel, Anderson Reis de Sousa

**Affiliations:** 1 Universidade Federal de Santa Catarina, Departamento de Enfermagem, Florianópolis, SC, Brazil.; 2 University of Ottawa, Faculty of Health Sciences, Ottawa, Canada.; 3 Universidade Federal da Bahia, Escola de Enfermagem, Salvador, BA, Brazil.

**Keywords:** Patient-Centered Care, Patient Participation, Health Strategies, Continuity of Patient Care, COVID-19

## Abstract

**Objective:**

To identify care coproduction strategies based on patient experience throughout the hospitalization process triggered by COVID-19.

**Method:**

This was a qualitative, exploratory-descriptive study derived from a multicenter research project conducted in Brazilian university hospitals. Thirty-four patients who were discharged after hospitalization for COVID-19 participated in the study. Data were collected from May 2021 to January 2022. The data were analyzed using thematic content analysis.

**Results:**

Thirty-two codes were generated, giving rise to the thematic category “Care coproduction in the pandemic setting”, composed of four subcategories: “Health education in care production”; “Care coproduction during hospitalization”; “Hospital discharge instructions and guidance following COVID-19”; and “Continuity of care during post-COVID-19 Recovery”.

**Conclusion:**

The findings revealed emerging strategies for care coproduction, including promoting health education, considering the patient’s experience and care journey during healthcare delivery, developing and implementing a shared discharge plan, and ensuring social support and access to the healthcare system throughout the health-disease-rehabilitation process.

## INTRODUCTION

The global crisis caused by COVID-19 not only led to a collapse in public health but also prompted the reassessment of healthcare systems, care models, and the quality of care provided^(1)^. In hospital settings, marked by prolonged hospitalizations and the need for intensive care during the pandemic, patient experience assumed a strategic role in the reorganization of healthcare services^(2)^.

Patient experience encompasses perceptions shaped through interactions established throughout the care process, reflecting, among other aspects, the organizational culture of healthcare services^(3)^. In this context, care coproduction stands out as the active collaboration among healthcare stakeholders (healthcare professional-patient-family), involving the sharing of knowledge, skills, and decision-making throughout the care process^(4,5)^.

In hospital settings, particularly in highly complex situations such as COVID-19, care coproduction has emerged as a key factor in improving clinical outcomes by strengthening relationships among healthcare professionals, patients, and families, while increasing engagement in care^(4,5)^. However, its effective implementation requires changes in institutional dynamics, encouraging patient participation and the sharing of information in an accessible manner by healthcare teams^(5,6)^.

The pandemic imposed major challenges to the operationalization of care coproduction, including restrictions on interpersonal contact, healthcare service overload, limited access to healthcare resources, social inequalities, and communication barriers, the effects of which extended beyond the critical period^(7,8,9)^. This situation highlighted the need to adapt care strategies to ensure continuity of care even in the face of adversity.

Despite advances in discussions on patient experience and care coproduction, there remains a gap in understanding the strategies that emerged from the experiences of patients hospitalized for COVID-19, particularly in the Brazilian context. Investigating these strategies is relevant to support the improvement of care practices and strengthen more participatory and resilient models of care.

Accordingly, this study was guided by the following research question: What care coproduction strategies emerged from patient experience during hospitalization for COVID-19? The objective was to identify care coproduction strategies based on patient experience throughout the hospitalization process triggered by COVID-19.

## METHOD

### Study Design

This was an exploratory-descriptive study with a qualitative approach^(10)^, derived from the master’s dissertation project entitled “Patient experience related to nursing care during hospitalization for COVID-19”, linked to the larger research project “Assessment of nursing care provided to patients with COVID-19 in Brazilian university hospitals”, whose protocol was approved by the Research Ethics Committee involving Human Beings under Opinion 4,347,463/2020 and Certificate of Presentation for Ethical Consideration 38912820.3.1001.0121.

### Setting

The study was conducted in wards/units of three federal university hospitals (FUHs) located in the Southern and Southeastern regions of Brazil, where nursing professionals provided care to individuals with suspected or confirmed COVID-19. These institutions were intentionally selected because they were recognized as referral centers for the care of patients with COVID-19, considering the affinity among the research groups and investigators, geographical proximity, and the different realities/experiences in coping with COVID-19.

### Population, Selection Criteria, and Sample Definition

The study included 34 patients who had recovered from the clinical condition caused by COVID-19 and had been discharged from the inpatient ward/unit of the HUFs. The number of participants was intentionally defined, and the sample size was determined according to the data saturation criterion^(11)^. Participants were randomly selected based on the institutions’ monthly hospital discharge lists. One out of every three individuals was contacted by telephone, starting with the first person on the list, and this procedure was maintained until data saturation was achieved.

The inclusion criteria were: (a) age over 18 years; (b) fluency in Brazilian Portuguese; and (c) hospitalization due to COVID-19 for at least 72 hours (clinical outcome = hospital discharge). The exclusion criteria were: (a) inability to provide informed consent—advanced disease, use of sedation, or neurological and psychiatric disorders; and (b) debilitated and/ or distressed individuals—respiratory distress, pain, immediate postoperative period, among others (conditions assessed during the initial telephone contact with the support of family members/caregivers)^(12)^.

### Data Collection

Data were collected from May 2021 to January 2022 by a research team composed of PhD- and master’s-level nurses and other healthcare professionals, as well as undergraduate and graduate nursing students, none of whom had a direct relationship with participants. After receiving prior training through workshops, the researchers conducted remote interviews by telephone, following the “*Manual para abordagem na entrevista de pacientes por meio da técnica de incidente crítico*”, with the aim of standardizing interview procedures and supporting the interview process with complementary information^(12)^.

The Critical Incident Technique^(13)^ enabled the identification of remarkable events experienced throughout hospitalization, allowing the capture of “situations”, “behaviors”, and “consequences” (positive or negative) relevant to understanding the patient experience in healthcare. These narrative elements allowed for richer descriptions of critical incidents, highlighting the difficulties encountered and revealing care coproduction strategies based on participants’ experiences.

The interviews were conducted using a script containing instructions for the remote approach, accompanied by a semi-structured instrument including questions related to the research topic, participants’ characterization data, a flowchart, and interview guidelines^(12)^. During data collection, the researchers adopted a qualified, empathetic, and nonjudgmental listening approach, recognizing participants’ narratives as central to understanding the phenomenon.

It is acknowledged that, due to their professional training and experience in the healthcare field, the researchers could have influenced data generation and interpretation. To mitigate this effect, continuous reflexivity was adopted throughout all stages of the study through team discussions, shared critical analysis, and explicit acknowledgment of the researchers’ assumptions, aiming to minimize potential bias and enhance the trustworthiness of the interpretations.

Subsequently, the interview data were audio-recorded (mean interview duration = 19 minutes) and manually transcribed verbatim using Microsoft Office Word^®^. A text refinement process was then performed, including the removal of incomprehensible or inappropriate words. Participants’ characterization data were recorded immediately during the telephone interview using a structured electronic form created in Google Forms^®^. Subsequently, these data were organized in Google Sheets^®(12)^.

### Data Analysis and Processing

Data were analyzed using thematic content analysis^(14)^, with the findings discussed based on the theoretical frameworks of patient experience^(3)^ and care management^(15)^, following these stages: (a) pre-analysis (data organization); (b) material exploration; and (c) treatment of the results (content inference and interpretation)^(14)^. During the pre-analysis stage, the interviews included in this study were read and reviewed, together with a review of the theoretical foundations that guided the data analysis. In addition, a horizontal reading of the material was performed, allowing the identification of the central idea underlying the interviews.

During the material exploration stage, the interviews were imported into NVivo 10^®^ (Windows^®^), where excerpts were selected and coded using words or groups of words to summarize their content. Simultaneously, a spreadsheet was created in Microsoft Excel^®^ to provide an overview of the codes generated in the software, facilitating the identification of similarities, differences, and connections among them.

**Figure 1:**
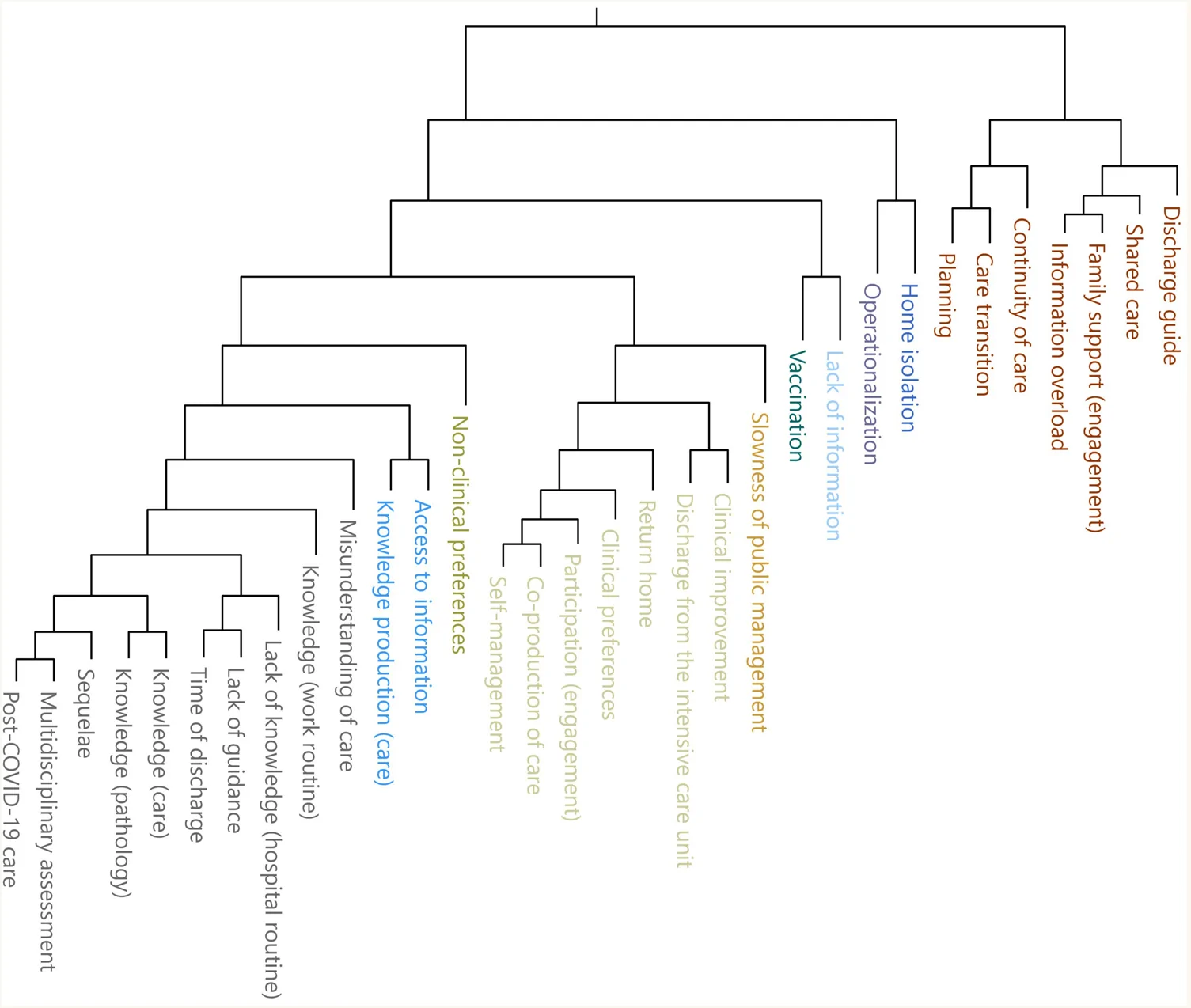
Clustered codes based on word similarity – Florianópolis, Santa Catarina, Brazil, 2026.

During the processing of results, content inference, and interpretation, 32 codes were generated. Analysis of these elements using the clustering tool available in NVivo 10^®^ (Windows^®^) enabled the construction of a vertical dendrogram, as illustrated in Figure 1. This tree-structured graphical representation made it possible to identify the relationships of proximity and similarity among the generated codes.

Based on this process, the codes were reorganized both in the software and in the Microsoft Excel^®^ spreadsheet according to the semantic relationships among the words, giving rise to the thematic category of interest and its respective subcategories. These were subsequently interpreted in light of the adopted theoretical frameworks. The categorization performed in the software enabled the analysis of 114 interview excerpts, comprising 241 references associated with the identified codes. It is noteworthy that some excerpts and certain codes encompassed more than one thematic subcategory because of the interrelationship among the contents expressed in the interviews.

To strengthen the analytical trustworthiness of the findings, the analytical process was conducted iteratively through repeated readings of the empirical material, allowing continuous refinement of the codes and emerging categories to ensure internal consistency and adherence to the data. To ensure the credibility of the findings, the constructed categories were continuously compared with the empirical material to confirm their representativeness and fidelity to participants’ narratives. Finally, the findings underwent a critical and reflective review by the research team, promoting deeper interpretation and theoretical-methodological alignment.

### Ethical Aspects

This study complies with the guidelines established by Circular Letter 2, issued on February 24, 2021, by the Brazilian National Research Ethics Commission^(16)^, and Resolution 466, issued on December 12, 2012, by the Brazilian National Health Council^(17)^, which regulate research involving human participants. Participants’ rights were ensured through an Informed Consent Form, which was read during the telephone call. Confidentiality was guaranteed, and participation was voluntary and without financial compensation. Verbal consent was audio-recorded. The study objectives and methods were explained beforehand. Participants were identified by the letter “P”, followed by their hospital admission code, consisting of one letter and three digits.

It is important to note that no artificial intelligence resources were used in generating the data for this study. However, ChatGPT 4.0^®^ was used exclusively for preliminary spelling and grammar corrections, while fully preserving the originality of the manuscript and the authors’ ideas, which are properly referenced throughout the text^(18)^. Furthermore, to ensure methodological rigor, this study followed the recommendations of the COnsolidated criteria for REporting Qualitative research^(19)^.

## RESULTS

The study included 34 participants, of whom 52.9% (*n* = 18) were men and 47.1% (*n* = 16) were women, with a mean age of 53 years (range: 21–82 years). Concerning educational attainment, 17.6% (*n* = 6) had completed higher education, 41.2% (*n* = 14) had completed high school, 17.6% (*n* = 6) had completed the early years of elementary school, 20.6% (*n* = 7) had completed the final years of elementary school, and 3% (*n* = 1) reported no formal education or less than one year of schooling. The duration of hospitalization due to COVID-19 ranged from 3 to 90 days, with a mean length of stay of 21 days.

**Figure 2:**
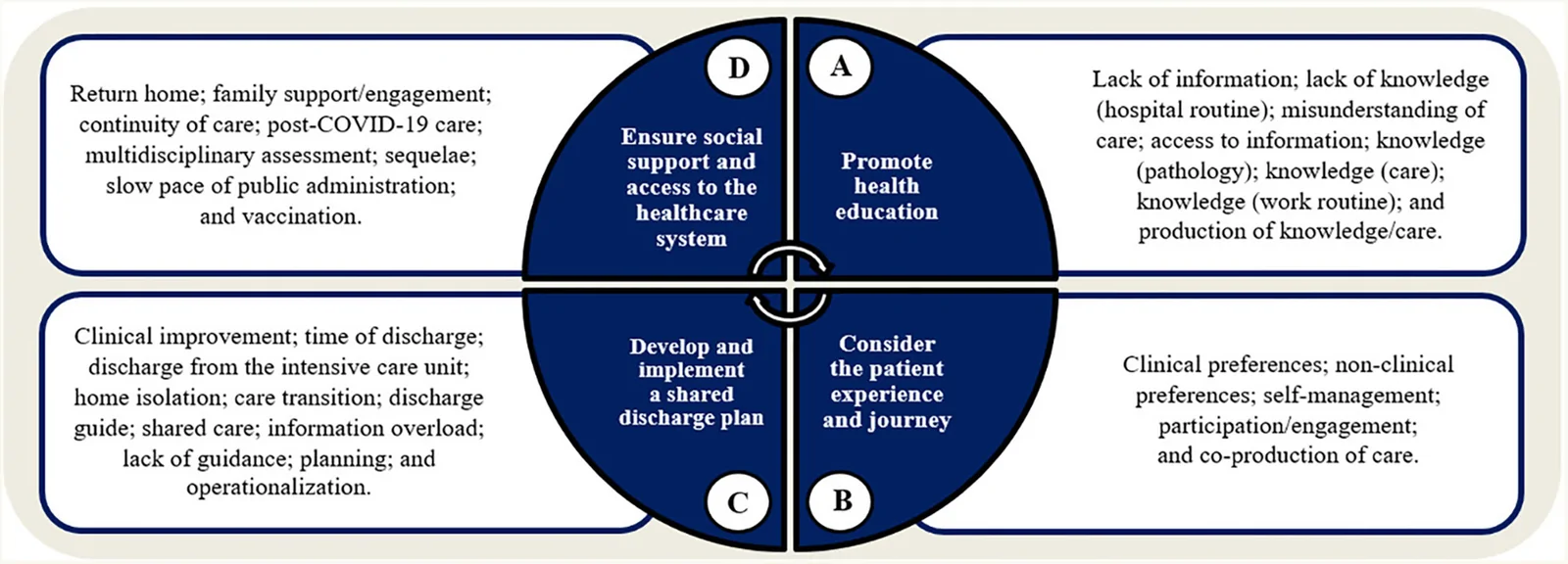
Contextualization of the set of codes linked to care coproduction strategies – Florianópolis, Santa Catarina, Brazil, 2026.

Following data organization and classification, the thematic category “Care coproduction in the pandemic setting” was identified, comprising four subcategories (groups of codes): (A) Health education in care production; (B) Care coproduction during hospitalization; (C) Hospital discharge instructions and guidance following COVID-19; and (D) Continuity of care during post-COVID-19 recovery. Figure 2, presented below, provides a visual representation of these four groups, highlighting the interconnections among the codes and the breadth and complexity of the care coproduction strategies identified in the study.

Within the first group of codes, “Health education in care production”, qualified access to information emerged as extending beyond the mere communicative dimension, constituting a structural element of care coproduction. Reports from users of the FUHs indicated that understanding their clinical condition, the procedures performed, and institutional routines contributed to more predictable care, the development of therapeutic relationships, and more collaborative interactions between patients and healthcare professionals. Conversely, situations characterized by a lack of information were associated with misunderstanding and detachment from the care received.

[...] *I was hospitalized for only nine days and just needed supplemental oxygen; I didn’t need to be intubated. That’s why I recovered relatively quickly, but I have underlying conditions, and COVID-19 is opportunistic—it targets the ailments you already have alongside the infection. Doctors had explained all of this to me before I contracted COVID-19* [...] (PD687).

[...] *everyone was very attentive* [...], *explaining what they were doing the whole time. One memorable aspect was the arterial blood gas test—mostly because of the pain involved—but it was nothing out of the ordinary. I was already familiar with the procedures, so the care I received didn’t come as a surprise to me* (PD205).

[...] *if a nursing technician was assigned to me* [...] *and the one assigned to another patient wasn’t doing anything* [...], *he wouldn’t come to help me even if I called him, simply because he wasn’t the one assigned to me. I find that terrible* [...] (PC1017).

These findings suggest that information functions as a mechanism for redistributing knowledge and decision-making power within the care setting, creating conditions for patient engagement throughout the hospitalization process. Furthermore, health education appears to provide the foundation for the other themes identified in this study, since active participation during hospitalization, understanding discharge recommendations, and continuity of care at home all depend, to varying degrees, on individuals’ ability to understand and apply knowledge related to their own health.

However, the findings also demonstrate that, although health education is necessary, it was not sufficient to ensure effective care coproduction. This aspect is evidenced by the subcategory “Care coproduction during hospitalization”, which reveals that patient involvement is directly related to recognition of their uniqueness, their clinical and non-clinical preferences, and their ability to exercise some degree of autonomy, all of which are essential for the development of self-management practices, even in situations of severe illness.

Participants’ accounts reinforce that seemingly routine actions, such as deciding how to perform personal hygiene or participating in oxygen management according to medical guidance, become meaningful because they represent concrete opportunities for negotiation and cooperation in care. Conversely, tensions emerged between institutional demands for standardized care practices and the participants’ desire to exercise greater autonomy in their own decisions. In specific situations, even when patients expressed a willingness to perform activities independently, healthcare professionals tended to adopt more protective approaches or practices guided by the traditional biomedical model.

[...] *I was already in the ward when they came to help me wash up in bed* [...], *but they used cold water. There was no warm water! I can’t stand cold water* [...]; *the water has to be lukewarm, even when it’s hot out* [...]*, so I would wash myself on my own—just a quick sponge bath every day before they arrived. I’d get up* [...] *and wash up, change my clothes, wash my panties, and hang them behind the bed* [...] (PD687).

[...] *often, regarding the oxygen, one person would come in, check the settings, and turn it up* [...], *then another would come and turn it down* [...], *even though the doctor had already said I should be allowed to wean myself off the* oxygen [...] (PA914).

[...] *they treated me very well. Little by little, they helped me and got me up. The only thing is, I don’t like being a burden; I prefer doing things for myself. That’s when I asked the nurses, “Could you let me wash up on my own?”* [...]; *however, they still kept helping me* [...] (PC307).

These findings corroborate that shared care within the FUHs did not occur in a linear manner but was shaped by ongoing negotiations involving patient autonomy, patient safety, and work organization. From this perspective, a positive hospitalization experience may be related not only to the technical quality of the interventions but also to the institutions’ ability to welcome and incorporate patients’ values, expectations, and preferences, as well as to promote their participation in care-related decision-making.

It is noteworthy that the “conflict” between patient participation and institutional control became even more evident within the FUHs at the time of hospital discharge. The elements comprising the group of codes “Hospital discharge instructions and guidance following COVID-19” indicate that discharge represents a particularly sensitive transition for maintaining patient engagement in treatment. Although participants reported positive feelings associated with returning home, their narratives reveal that the psycho-emotional and cognitive dimensions of this moment coexist with important communication and organizational weaknesses.

[...] *It was really good* [...] *my doctors stopped by in the morning and said, “Ready to go home?”* [...] *and I said, “Oh my God, that’s wonderful!” It was such a huge joy that my heart was pounding* [...] (PC1075).

[...] *I came home. Then he said* [...] *to take the necessary precautions, like staying isolated in my room* [...] *my daughter took care of me; she has a suitable place* [...] *I stayed the required number of days (*in isolation) *and, thank God, I didn’t have any complications. The only complication I’ve had since then was hair loss* (PC1038).

[...] *oh, they hardly said anything. They just mentioned that it’s not easy and that I had to take care of myself* [...] *then, when I was feeling better* [...] *I asked the doctor, “Doctor, am I going home soon?” So, I came home and started taking care of myself there* [...] *and things turned out fine* (PA896).

The difficulty participants experienced in recalling the instructions they had received, the absence of educational support materials, and the limited involvement of family members in the educational process reveal challenges that extend beyond the individual sphere and reflect the way healthcare services organize discharge planning. Thus, a direct relationship can be observed between this subcategory and the findings related to health education, since the effectiveness of discharge guidance depends not only on the transmission of information but also on the development of strategies that facilitate its understanding and incorporation into daily life at home.

[...] *I think they should call a family member in and explain the necessary care, because the people at home have no idea. They assume the person will come home exactly as they left* [...] *so you get home, and the family just “receives” you* [...] *I had a memory lapse and didn’t know how to explain or carry out the necessary care—things like the pureed diet, the period of avoiding exercise* [...] *we leave the hospital and don’t know what to do! The doctor gave me a lot of information, but by the time I stepped outside, I couldn’t remember it anymore. It’s so much information* [...] *maybe a pamphlet or a manual with some details for the family would help* [...] *because, for instance, I... since I developed diabetes after pancreatitis... I take insulin, and my family needs to know how to administer it and how to measure the dosage* [...] (PA895).

In this context, the proposal for an educational booklet or a shared discharge plan emerged as a response to the gaps identified by users of FUHs, reinforcing that the need for such a resource goes beyond simply providing recommendations and is closely related to care coordination across different settings and among different healthcare stakeholders. Considering tools capable of engaging patients and family members throughout this process reinforces that care coproduction encompasses not only interpersonal interactions but also resources designed to provide guidance and ensure continuity of care after hospital discharge.

In turn, the subcategory “Continuity of care during post-COVID-19 recovery” broadens the understanding of the phenomenon by extending beyond the institutional boundaries of the hospital environment and confirming that its sustainability depends on both social and organizational factors. The participants’ reports demonstrate that coping with the physical and emotional sequelae of COVID-19 required substantial involvement from the participants and their support networks, as well as timely access to different healthcare services and professionals.

[...] *so, on Tuesday, I was discharged. The nurse took me to the bathroom in the morning* [...] *and I noticed some chafing—I think it was because I hadn’t been cleaned properly. When I got home and went to shower* [...] *wow! I even remarked, “I came home to shower, but it was such a struggle* [...] *it felt like everything was oozing* [...] *all that dirty, stuck-on mess”. I felt horrible* [...] (PD736).

[...] *I still experience shortness of breath to this day; it hinders me a bit, but according to what the doctor told me, it’s still a lingering effect of COVID-19* [...] *so, I’m doing physical therapy on Tuesdays and Thursdays; I’ve even brought the exercises home* [...] *I also saw a psychiatrist* [...] *because at night, I would cry a lot and couldn’t sleep* (PD687).

[...] *I needed an appointment with a pulmonologist after I’d already been discharged* [...], *and that was the part that took a little while* [...] *getting that appointment scheduled* [...] (PD535).

The delay in obtaining specialized consultations and the need for multidisciplinary follow-up demonstrate that patients’ active participation in restoring their own health is conditioned by the healthcare system’s ability to provide timely support and respond appropriately to the demands that arise throughout the health-disease-rehabilitation process. Although care coproduction presupposes patient engagement, its implementation is strongly associated with the conditions that facilitate or limit the exercise of this active role, highlighting the interdependence between actions developed at the micro-level of care and the macrostructural elements related to healthcare delivery.

Together, the four subcategories portrayed distinct stages of the patient journey during the pandemic and revealed that care coproduction within the FUHs was a dynamic, continuous, and relational phenomenon, constructed through the interaction among patients, family members, healthcare professionals, and healthcare services. Furthermore, the findings confirm that effective care coproduction simultaneously involves valuing the patient experience, improving communication practices, organizing the transition between hospital and home settings, and ensuring the healthcare system’s commitment to maintaining continuity of care.

## DISCUSSION

The main conceptual contribution of this study lies in understanding care coproduction as a longitudinal, multilevel, and multidimensional process that integrates experiences, knowledge, and resources distributed throughout the patient’s journey within the healthcare system, rather than as something that occurs only during specific interactions among healthcare professionals, patients, and family members. During the COVID-19 pandemic, this process unfolded under conditions characterized by vulnerability, clinical uncertainty, and healthcare service reorganization, requiring different forms of communication, participation, and shared responsibility among the stakeholders involved in healthcare delivery.

In this context, the availability and quality of information emerged as key drivers of care coproduction strategies. Access to knowledge is a fundamental component of health education and the development of knowledge within institutions and communities, fostering more informed decisions regarding health and treatment^(20)^. Studies have highlighted the need to provide guidance regarding the procedures performed during hospitalization and indicate that clear and accessible information may help reduce fear and anxiety, particularly during public health crises^(21,22)^. Furthermore, transparent communication between healthcare professionals and patients promotes trusting relationships, empowers patients, and expands opportunities for active participation in care^(22)^.

However, providing understandable guidance, although necessary, is not sufficient by itself to achieve care coproduction. It is equally important to consider patients’ perceptions, experiences, and individual preferences throughout hospitalization and during the transition to home^(23)^. This perspective aligns with the principles of patient-centered care by recognizing patients’ autonomy, respecting their dignity, and promoting their engagement in treatment-related decision-making while seeking to understand their concerns, beliefs, values, and preferences^(24,25)^. In this way, self-management strategies and coproduction among the different healthcare stakeholders involved in care are enabled.

The literature indicates that the relationship between patient-centered care and patient experience is reflected in coproduction practices, emphasizing the role of healthcare professionals in assessing each patient’s profile to provide holistic and safe care aimed at achieving excellence throughout the patient journey within healthcare institutions^(26,27)^. However, the coproduction approach focuses on establishing a cocreation partnership between the provider and the service user, at the “micro” level of value creation, recognizing that care management is not limited to internal interventions and care techniques but encompasses the individual’s overall experience throughout the health-disease-care process^(27)^.

This understanding reinforces the importance of discharge instructions and guidance for care transition and for its shared development during the COVID-19 pandemic, highlighting the potential of structured tools, such as discharge plans or educational booklets, tailored to patients’ needs and the specific characteristics of the disease^(28)^. Likewise, the inclusion of patients and family members in clinical and care-related discussions, together with the multidisciplinary team, from admission through discharge, may benefit the shared decision-making process and contribute to care coproduction and continuity of care after hospitalization^(25,27)^.

Consistent with this discussion, a study conducted between February and November 2021 involving 300 patients recovering from COVID-19 who were discharged from a university hospital in Southern Brazil reported a positive assessment of the hospital-to-home transition among survivors, although areas requiring improvement were identified, particularly with regard to discharge/care planning and post-hospitalization referrals. Conversely, caregivers and family members expressed a less favorable perception, attributed to the difficulties encountered after discharge, highlighting weaknesses in care transition and the importance of involving them in discharge planning before hospital discharge in order to improve its implementation and ensure continuity of care in cases of COVID-19^(29)^.

Another aspect that deserves attention concerns the adversities imposed by the pandemic on maintaining and jointly constructing care in the home setting, highlighting the importance of access to healthcare services and social support during recovery and rehabilitation. The rapid evolution of the public health crisis, together with the frequent changes in care recommendations and organizational limitations within healthcare services, affected care coproduction and the continuity of care provided to patients and their families^(30)^. At the same time, the pandemic highlighted and, in many cases, exacerbated pre-existing socioeconomic inequalities, influencing access to the resources required for treatment and health recovery^(8,9)^.

From this perspective, care coproduction requires coordinated strategies at the clinical, managerial, and health system levels that are capable of considering both individuals’ unique characteristics and the collective determinants that shape patient experience throughout the health-disease-rehabilitation process, with the aim of redesigning, coordinating, improving, and ensuring continuity of care across the different levels of healthcare delivery^(23,28,29)^. Therefore, it is essential to deepen the discussion regarding the need for resilient care models and healthcare systems capable of responding to critical situations, strengthening primary and community care, incorporating innovative technologies into care management, and integrating coproduction practices into both clinical practice and nursing and healthcare management.

Considering the role of nursing in coordinating care activities, this study indicates that the profession occupies a strategic position in operationalizing care coproduction, since continuous proximity to patients and family members facilitates the identification of needs and difficulties experienced throughout hospitalization, enabling the development of educational and relational interventions focused on patients’ active participation in care. From the perspective of care management, this discussion reinforces the importance of institutional initiatives aimed at strengthening care coproduction practices, including structured care transition protocols, educational materials tailored to patients’ realities, and improvements in communication processes.

Among the study limitations, the remote interviews may have influenced the depth of the narratives and the understanding of certain experiences related to hospitalization for COVID-19. In addition, technical difficulties during interview recording and memory lapses reported by some participants may have limited the recall of certain care-related events. Nevertheless, the adopted strategy enabled access to geographically dispersed participants and allowed the understanding of experiences across different healthcare settings throughout the pandemic.

## CONCLUSION

Based on the findings, relevant care coproduction strategies for the post-pandemic context were identified, including promoting health education so that patients and family members can make informed decisions; valuing the patient’s experience and journey so that care is centered on their needs; collaboratively developing discharge plans involving healthcare professionals, patients, and family members to ensure continuity of care; and ensuring social support and access to the healthcare system throughout the health-disease-rehabilitation process. The findings also highlight the importance of developing professional competencies related to qualified listening, shared decision-making, and recognition of the patient experience. Considering the qualitative design and the investigated setting, the results provide theoretical support for improving care practices, without intending to generalize the findings to other contexts.

## Data Availability

The dataset supporting the findings of this study is available in SciELO Data and can be accessed at: https://doi.org/10.48331/SCIELODATA.WLPY5A.
