# Supplementary material for: Care coproduction strategies based on the experience of patients hospitalized for COVID-19: a multicenter qualitative study
Source: Rev Esc Enferm USP. 2026 Sep 18;60:e20250354. doi: 10.1590/1980-220X-REEUSP-2025-0354en (PMC13600733; doi:10.1590/1980-220X-REEUSP-2025-0354en)
Supplement: Translation

# Translation: Estratégias de coprodução do cuidado a partir da experiência de pacientes hospitalizados pela COVID-19: estudo qualitativo multicêntrico*

**DOI:** 10.1590/1980-220X-REEUSP-2025-0354pt

**Authors:** Jhonata de Souza Joaquim, Diovane Ghignatti da Costa, Alacoque Lorenzini Erdmann, Franciely Daiana Engel, Anderson Reis de Sousa

**Affiliations:** 1 Universidade Federal de Santa Catarina, Departamento de Enfermagem, Florianópolis, SC, Brasil.; 2 University of Ottawa, Faculty of Health Sciences, Ottawa, Canada.; 3 Universidade Federal da Bahia, Escola de Enfermagem, Salvador, BA, Brasil.

**Keywords:** Assistência Centrada no Paciente, Participação do Paciente, Estratégias de Saúde, Continuidade da Assistência ao Paciente, COVID-19

## Abstract

**Objetivo:**

Identificar as estratégias de coprodução do cuidado com base na experiência do paciente ao longo do processo de hospitalização desencadeado pela COVID-19.

**Método:**

Trata-se de estudo qualitativo, exploratório-descritivo, oriundo de pesquisa multicêntrica realizada em hospitais universitários brasileiros. Participaram 34 pacientes que receberam alta após internação pela COVID-19. A coleta de dados ocorreu de maio de 2021 a janeiro de 2022. Os dados foram tratados por meio da análise de conteúdo temática.

**Resultados:**

Foram gerados 32 códigos, os quais originaram a categoria temática “Coprodução de cuidados no cenário pandêmico”, composta por quatro subcategorias: “Educação em saúde na produção do cuidado”; “Coprodução de cuidados durante a internação hospitalar”; “Instruções e orientações de alta hospitalar decorrente da COVID-19”; e “Continuidade do cuidado na recuperação pós-COVID-19”.

**Conclusão:**

Os achados da pesquisa revelaram estratégias emergentes para a coprodução do cuidado, incluindo promover a educação em saúde, considerar a experiência e a jornada do paciente durante a assistência, desenvolver e implementar um plano de alta compartilhado, e garantir apoio social e acesso ao sistema de saúde no processo de saúde-doença-reabilitação.

## INTRODUÇÃO

A crise global provocada pela COVID-19 não apenas gerou um colapso na saúde pública, mas também impulsionou a reavaliação dos sistemas de saúde, dos modelos assistenciais e da qualidade do cuidado ofertado^(1)^. No âmbito hospitalar, marcado por internações prolongadas e pela necessidade de cuidados intensivos durante a pandemia, a experiência do paciente passou a assumir um papel estratégico na reorganização dos serviços de saúde^(2)^.

A experiência do paciente compreende percepções construídas a partir das interações estabelecidas ao longo do cuidado, refletindo, inclusive, os aspectos da cultura organizacional dos serviços^(3)^. Nesse contexto, destaca-se a coprodução do cuidado em saúde, entendida como a colaboração ativa entre os atores da saúde (profissional-paciente-familiar), com compartilhamento de conhecimentos, habilidades e decisões no processo assistencial^(4,5)^.

No cenário hospitalar, especialmente em situações de maior complexidade, como a COVID-19, a coprodução do cuidado se configura como um elemento potencializador de melhores desfechos clínicos, ao fortalecer a relação entre profissionais, pacientes e familiares, e ampliar o engajamento no cuidado^(4,5)^. Contudo, sua efetivação requer mudanças na dinâmica institucional, com incentivo à participação dos usuários e ao compartilhamento de informações de forma acessível pelas equipes de saúde^(5,6)^.

A pandemia impôs importantes desafios à operacionalização dessa coprodução, como restrições de contato, sobrecarga dos serviços, limitações no acesso aos recursos de saúde, desigualdades sociais e dificuldades comunicacionais, cujos efeitos se estenderam além do período crítico^(7,8,9)^. Tal situação evidenciou a necessidade de adaptação das estratégias de cuidado, de modo a assegurar sua continuidade, mesmo diante de adversidades.

Apesar do avanço das discussões sobre a experiência do paciente e a coprodução do cuidado, observa-se uma lacuna quanto à compreensão das estratégias que surgiram da vivência de pacientes hospitalizados pela COVID-19, especialmente no contexto brasileiro. Investigar essas estratégias se torna relevante para subsidiar a qualificação das práticas assistenciais e o fortalecimento de modelos de cuidado mais participativos e resilientes.

Diante disso, este estudo foi guiado pela seguinte questão de investigação: quais estratégias de coprodução do cuidado em saúde emergiram da experiência do paciente durante a internação pela COVID-19? Como objetivo, busca-se identificar as estratégias de coprodução do cuidado com base na experiência do paciente ao longo do processo de hospitalização desencadeado pela COVID-19.

## MÉTODO

### Tipo do Estudo

Trata-se de um estudo exploratório-descritivo, de abordagem qualitativa^(10)^, derivado do projeto de dissertação de mestrado intitulado “Experiência do paciente relacionada ao cuidado de enfermagem durante a internação hospitalar por COVID-19”, vinculado ao macroprojeto “Avaliação do cuidado de enfermagem a pacientes com COVID-19 em hospitais universitários brasileiros”, cujo protocolo foi aprovado pelo Comitê de Ética em Pesquisa com Seres Humanos, sob o Parecer nº 4.347.463/2020 e o Certificado de Apresentação para Apreciação Ética nº 38912820.3.1001.0121.

### Local

O cenário deste estudo é composto por setores/unidades de três hospitais universitários federais (HUFs), situados nas regiões Sul e Sudeste do Brasil, nos quais os profissionais de enfermagem desempenharam atividades de assistência a indivíduos com suspeita ou diagnóstico de COVID-19. Tais instituições foram escolhidas por serem reputadas como referência no cuidado de pacientes com a COVID-19, sendo selecionadas de forma intencional, considerando a afinidade entre os grupos de pesquisa e pesquisadores, a proximidade geográfica e as diferentes realidades/experiências no enfrentamento da COVID-19.

### População, Critérios de Seleção e Definição da Amostra

Participaram do estudo 34 pacientes recuperados do quadro clínico desencadeado pela COVID-19, que receberam alta do setor/unidade de internação hospitalar dos HUF. O número de participantes foi definido intencionalmente, e a amostra foi determinada pelo critério de saturação dos dados^(11)^. A seleção dos participantes ocorreu de maneira aleatória, com base na lista mensal de altas das instituições. Foi realizado contato telefônico com um a cada três indivíduos, iniciando pelo primeiro da lista, mantendo esse procedimento até alcançar a saturação.

Os critérios de inclusão adotados foram: a) idade superior a 18 anos; b) fluência no idioma português brasileiro; e c) internação ocasionada pela COVID-19 por no mínimo 72 horas (desfecho clínico = alta hospitalar). Constituíram os critérios de exclusão: a) falta de capacidade para emitir consentimento – doença avançada, uso de sedação, e alterações neurológicas e psiquiátricas; e b) indivíduos debilitados e/ou angustiados – dificuldade respiratória, dor, pós-operatório imediato, entre outros (condições verificadas no primeiro contato telefônico, com o apoio de familiares/cuidadores)^(12)^.

### Coleta de Dados

Os dados foram coletados de maio de 2021 a janeiro de 2022 por uma equipe de pesquisa composta por doutores e mestres em enfermagem e em áreas da saúde, além de discentes de graduação e pós-graduação em enfermagem, sem vínculo direto com os participantes. Previamente capacitados por meio de oficinas, os pesquisadores realizaram entrevistas remotas, via ligação telefônica, orientadas pelo “Manual para abordagem na entrevista de pacientes por meio da técnica de incidente crítico”, com o intuito de padronizar a condução e subsidiar a abordagem com informações complementares^(12)^.

A utilização da Técnica do Incidente Crítico^(13)^ possibilitou a identificação de eventos marcantes vivenciados ao longo da hospitalização, permitindo a apreensão de “situações”, “comportamentos” e “consequências” (positivas ou negativas) relevantes para a compreensão da experiência do paciente no cuidado em saúde. Esses elementos narrativos possibilitaram descrições mais densas dos incidentes críticos, evidenciando as dificuldades enfrentadas e revelando estratégias de coprodução do cuidado a partir das vivências relatadas.

As entrevistas foram conduzidas a partir de um roteiro com instruções para a abordagem remota, acompanhado de um instrumento semiestruturado contendo questões de interesse relacionadas ao objeto investigado, aos dados de caracterização dos participantes, a um fluxograma e às orientações pertinentes à entrevista^(12)^. Durante a coleta, os pesquisadores assumiram uma postura de escuta qualificada, empática e não julgadora, valorizando as narrativas dos participantes como centrais para a compreensão do fenômeno.

Reconhece-se que os pesquisadores, em razão de sua formação e de suas experiências na área da saúde, poderiam influenciar a produção e a interpretação dos dados. Para mitigar esse efeito, adotou-se a reflexividade contínua em todas as etapas do estudo, com discussões em equipe, análise crítica compartilhada e explicitação dos pressupostos dos pesquisadores, visando minimizar possíveis vieses e ampliar a fidedignidade das interpretações.

Posteriormente, os dados das entrevistas foram gravados em dispositivos de áudio (duração média das entrevistas = 19 minutos) e transcritos manualmente na íntegra por meio do processador de texto *Microsoft Office Word* ^®^. Realizou-se um processo de refinamento textual, que incluiu a exclusão de palavras incompreensíveis ou inapropriadas. Os dados de caracterização dos participantes foram registrados prontamente no momento da ligação telefônica, utilizando-se um formulário eletrônico estruturado no aplicativo de gerenciamento de pesquisas *Google Forms* ^®^. Em seguida, esses dados foram tabulados no programa de planilhas *Google Sheets* ^®(12)^.

### Análise e Tratamento dos Dados

Os dados obtidos foram analisados por meio da análise de conteúdo temático^(14)^, com a discussão dos achados fundamentada nos referenciais teóricos de experiência do paciente^(3)^ e gestão do cuidado^(15)^, seguindo as seguintes etapas: a) pré-análise (organização de dados); b) exploração do material; e c) tratamento dos resultados (inferência e interpretação do conteúdo)^(14)^. Na fase de pré-análise, realizou-se a leitura e a verificação das entrevistas incluídas neste estudo, acompanhadas por uma revisão dos fundamentos teóricos que orientaram a análise de dados. Além disso, efetuou-se uma leitura horizontal dos materiais, permitindo a compreensão da ideia central das entrevistas.

Na etapa de exploração do material, as entrevistas foram incorporadas ao *software NVivo 10* ^®^ (*Windows* ^®^), no qual trechos foram selecionados e códigos, representados por palavras ou conjuntos de palavras, foram criados para sintetizá-los. Simultaneamente, uma planilha foi elaborada no editor *Microsoft Excel* ^®^ para apresentar o panorama dos códigos obtidos no *software*, facilitando a identificação de similaridades, diferenças e conexões entre eles.

No tratamento dos resultados, na inferência e na interpretação do conteúdo, foram gerados 32 códigos. A análise desses elementos, realizada por meio da ferramenta de agrupamento (*cluster*) do *software NVivo 10* ^®^ (*Windows* ^®^), possibilitou a construção de um dendrograma vertical, conforme ilustrado na Figura 1. Essa representação gráfica, estruturada em forma de árvore, permitiu identificar as relações de proximidade e de similaridade entre os códigos produzidos.

**Figura 1:** Códigos em *cluster* por similaridade de palavra – Florianópolis, Santa Catarina, Brasil, 2026.

A partir desse processo, os códigos foram reorganizados tanto no *software* quanto na planilha do editor *Microsoft Excel* ^®^, de acordo com a semântica das palavras, originando a categoria temática de interesse e as suas respectivas subcategorias. Essas, por sua vez, foram interpretadas à luz dos referenciais teóricos adotados. A categorização no *software* oportunizou a análise de 114 trechos das entrevistas, com 241 referências associadas aos códigos identificados. Ressalta-se que alguns trechos e determinados códigos abarcaram mais de uma subcategoria temática, devido à inter-relação dos conteúdos presentes nas entrevistas.

No intuito de fortalecer a confiabilidade analítica dos achados, adotou-se a condução do processo analítico de forma iterativa, com leituras sucessivas do material empírico, possibilitando o refinamento contínuo dos códigos e das categorias emergentes, com vistas à coerência interna e à aderência aos dados. Com o objetivo de garantir a credibilidade dos resultados, as categorias construídas foram continuamente confrontadas com o material empírico, buscando assegurar sua representatividade e fidelidade às narrativas dos participantes. Por fim, procedeu-se à revisão crítica e reflexiva dos achados no âmbito da equipe de pesquisa, favorecendo o aprofundamento das interpretações e o alinhamento teórico-metodológico.

### Aspectos Éticos

Este estudo segue as diretrizes estabelecidas pelo Ofício Circular nº 2, de 24 de fevereiro de 2021, da Comissão Nacional de Ética em Pesquisa^(16)^ e da Resolução nº 466, de 12 de dezembro de 2012, do Conselho Nacional de Saúde^(17)^, que regulamentam pesquisas com seres humanos. Os participantes tiveram seus direitos assegurados por meio de um Termo de Consentimento Livre e Esclarecido, lido durante a ligação telefônica. A confidencialidade foi garantida, e a participação ocorreu de forma voluntária e sem fins lucrativos. O consentimento verbal foi registrado em áudio/gravação. Os objetivos e métodos da pesquisa foram explicados previamente. A identificação dos participantes foi realizada por meio da letra “P”, seguida do código de internação hospitalar, composto por uma letra e três algarismos.

Cabe destacar que não foram empregados recursos de inteligência artificial na geração dos dados desta pesquisa. No entanto, ressalta-se que foi utilizado o *ChatGPT 4.0* ^®^ exclusivamente para correções ortográficas e gramaticais preliminares, preservando-se integralmente a originalidade e as ideias do autor, devidamente referenciadas ao longo do texto^(18)^. Ademais, com o intuito de assegurar o rigor metodológico, este estudo segue as diretrizes estabelecidas pelo guia *COnsolidated criteria for REporting Qualitative research* ^(19)^.

## RESULTADOS

O estudo analisou 34 participantes, dos quais 52,9% (*n* = 18) eram homens e 47,1% (*n* = 16) eram mulheres, com média de idade de 53 anos (variação de 21 a 82 anos). Quanto à escolaridade, 17,6% (*n* = 6) tinham ensino superior, 41,2%, ensino médio (*n* = 14), 17,6% (*n* = 6), ensino fundamental (séries iniciais), 20,6% (*n* = 7), ensino fundamental (séries finais), e 3% (*n* = 1) relataram a ausência de instrução formal ou menos de um ano de estudo. A duração da internação hospitalar desencadeada pela COVID-19 oscilou entre 3 e 90 dias, com uma média de 21 dias.

A partir da organização e classificação dos dados, alcançou-se a categoria temática “Coprodução de cuidados no cenário pandêmico”, constituída por quatro subcategorias (conjuntos de códigos), denominadas: (A) Educação em saúde na produção do cuidado; (B) Coprodução de cuidados durante a internação hospitalar; (C) Instruções e orientações de alta hospitalar decorrente da COVID-19; e (D) Continuidade do cuidado na recuperação pós-COVID-19. A Figura 2, apresentada a seguir, oferece uma representação visual desses quatro grupos, ressaltando a interconexão entre os códigos e a abrangência/complexidade das estratégias de coprodução do cuidado identificadas no estudo.

**Figura 2:** Contextualização do conjunto de códigos vinculados às estratégias de coprodução do cuidado – Florianópolis, Santa Catarina, Brasil, 2026.

No primeiro conjunto de códigos, “Educação em saúde na produção do cuidado”, evidencia-se que o acesso qualificado à informação ultrapassa a dimensão meramente comunicacional, constituindo-se como elemento estruturante da coprodução do cuidado. Os relatos dos usuários dos HUFs indicam que a compreensão da condição clínica, dos procedimentos realizados e das rotinas institucionais favorece uma assistência mais previsível, a construção de vínculos e o desenvolvimento de relações mais colaborativas entre pacientes e profissionais. Em contrapartida, situações marcadas pela falta de informações foram associadas à incompreensão e ao distanciamento em relação à assistência recebida.

[...] *fiquei só nove dias internada e fiquei apenas no oxigênio. Não precisei ser intubada. Então, foi onde eu me recuperei mais rapidamente, mas tenho comorbidades e a COVID-19 é oportunista* [...] *aí, ela vai atingindo aquelas enfermidades que você já tem junto com ela. Antes de ter a COVID-19, tudo isso, os médicos me explicaram* [...] (PD687).

[...] *todos muito atenciosos* [...]*, explicavam o tempo todo o que estavam fazendo. Uma parte marcante foi a questão da gasometria, mais pela dor que se sente quando é feito, mas nada fora do comum. Eu já conhecia os procedimentos de cor, então para mim não houve “espanto” nos cuidados* (PD205).

[...] *se um técnico de enfermagem está comigo* [...] *e o outro que está com outro paciente não está fazendo nada* [...], *mesmo eu chamando-o, ele não vinha me ajudar, porque não era ele que estava comigo. Eu acho isso horrível* [...] (PC1017).

Esses resultados sugerem que a informação atua como mecanismo de redistribuição do conhecimento e do poder decisório no contexto assistencial, criando condições para o envolvimento dos pacientes ao longo do processo de hospitalização. Além disso, observa-se que a educação em saúde é um alicerce para as demais temáticas identificadas neste estudo, uma vez que a participação ativa durante a internação, a compreensão das recomendações de alta e a continuidade da assistência no domicílio dependem, em diferentes níveis, da capacidade das pessoas de assimilar e utilizar conhecimentos relacionados à própria saúde.

Entretanto, os achados também demonstram que a educação em saúde, embora seja necessária, não foi suficiente para assegurar a efetiva coprodução do cuidado. Tal aspecto é evidenciado pela subcategoria “Coprodução de cuidados durante a internação hospitalar”, que revela que o envolvimento dos usuários está diretamente relacionado ao reconhecimento de sua singularidade, de suas preferências clínicas e não clínicas e de sua destreza para exercer algum grau de autonomia, sendo determinante para o desenvolvimento de práticas de automanejo, mesmo em situações de adoecimento grave.

Os depoimentos reforçam que ações aparentemente cotidianas, como decidir sobre a própria higiene ou participar do manejo do oxigênio conforme orientação médica, assumem relevância por representarem oportunidades concretas de negociação e cooperação no cuidado. Por outro lado, emergem tensões entre as demandas institucionais de padronização das práticas assistenciais e o desejo dos participantes de exercer maior protagonismo em suas escolhas. Em circunstâncias específicas, mesmo quando manifestavam interesse em realizar atividades de forma independente, prevaleciam condutas profissionais mais protetivas ou orientadas pela lógica biomédica tradicional.

[...] *eu já estava na enfermaria quando elas fizeram uma higiene no leito* [...], *mas com água fria. Não tinha água morna! Eu não suporto água fria* [...]; *a água tem que estar em meio termo, mesmo estando calor* [...] *aí, eu sozinha me lavava, era um “banhinho de gato” todo dia, antes delas chegarem. Eu levantava* [...] *e já fazia minha higiene, trocava a minha roupinha, lavava a minha calcinha e botava atrás da cama* [...] (PD687).

[...] *muitas vezes, ali no oxigênio, um entrava, botava o dedo e aumentava* [...], *o outro vinha e diminuía* [...], *porém a médica já tinha dito que era para deixar eu ir fazendo o desmame do oxigênio* [...] (PA914).

[...] *eles me trataram muito bem. Aos poucos, foram me auxiliando e me levantaram. A única coisa que eu posso dizer é que eu não gosto de dar trabalho; eu prefiro fazer as minhas próprias coisas. Foi aí que eu pedi aos enfermeiros: “Vocês poderiam me deixar fazer a minha própria higiene?”* [...]*; no entanto, eles ainda continuaram me auxiliando* [...] (PC307).

Essas evidências corroboram que o cuidado compartilhado nos HUFs não ocorreu de maneira linear, sendo permeado por negociações que abrangeram desde a autonomia do paciente até a segurança assistencial e a organização do trabalho. Nessa perspectiva, uma hospitalização positiva pode estar relacionada à qualidade técnica das intervenções, à capacidade das instituições de acolher e incorporar valores, expectativas e preferências dos pacientes, e à sua participação nos processos decisórios relacionados aos cuidados.

Destaca-se que o “conflito” entre participação e controle institucional se tornou ainda mais explícito nos HUFs no momento da alta hospitalar. Os elementos que compõem o conjunto de códigos “Instruções e orientações de alta hospitalar decorrentes da COVID-19” apontam que a alta representa uma transição particularmente sensível para a manutenção do envolvimento dos pacientes em seu tratamento. Embora os participantes tenham relatado sentimentos positivos associados ao retorno para casa, as narrativas expõem que a dimensão psicoemocional e cognitiva desse momento convive com importantes fragilidades comunicacionais e organizacionais.

[...] *foi muito bom* [...] *os meus médicos passaram de manhã e falaram: “Pronta para ir embora?”* [...] *aí, eu falei: “Ai, meu Deus, que maravilha!”. Foi uma alegria tão grande que o coração pulou demais* [...] (PC1075).

[...] *eu vim para casa. Aí, ele falou* [...] *para ter aqueles cuidados de ficar isolado no quarto* [...] *minha filha tomou conta de mim, ela tem um local adequado* [...] *eu fiquei os dias certos* (de isolamento) *e, graças a Deus, não tive complicação nenhuma. A única complicação que eu tive agora foi a queda de cabelo* (PC1038).

[...] *ah, eles não falavam quase nada. Só mencionavam que não é fácil e tinha que se cuidar* [...] *daí, quando eu estava melhor* [...] *eu perguntei ao médico: “Doutor, eu vou para casa logo?”. Então, eu vim para casa e comecei a me cuidar em casa* [...] *aí, ficou bom* (PA896).

A dificuldade de recordar as orientações recebidas, a ausência de materiais de apoio e a limitada inclusão dos familiares no processo educativo revelam desafios que extrapolam a esfera individual e remetem à forma como os serviços articulam o planejamento da alta. Assim, observa-se uma relação direta entre essa subcategoria e os resultados referentes à educação em saúde, uma vez que a efetividade das orientações depende não apenas da transmissão de informações, mas também da elaboração de estratégias capazes de favorecer sua compreensão e incorporação ao cotidiano domiciliar.

[...] *acho que deveriam chamar alguma pessoa da família e explicar os cuidados, porque as pessoas que estão em casa não têm noção. Elas acham que a pessoa vai chegar em casa como ela saiu* [...] *aí, você chega em casa e a família “só recebe”* [...] *eu tive um lapso de memória e não sabia como explicar e realizar os cuidados necessários, como, por exemplo, a alimentação pastosa, o período sem praticar algum exercício* [...] *a gente sai de lá e não sabe o que fazer! O médico me deu uma série de informações, mas quando cheguei na rua, já não lembrava mais. São muitas informações* [...] *talvez, uma cartilha ou um manual com algumas informações para a família* [...] *pois, por exemplo, eu* [...] *por ter desenvolvido diabetes após uma pancreatite* [...] *“tomo” insulina e a família precisa saber como aplicar, como medir* [...] (PA895).

Nesse cenário, a proposta de uma cartilha ou de um plano de alta compartilhado emergiu como resposta às lacunas apontadas pelos usuários dos HUFs, reforçando que a necessidade desse recurso perpassa a simples oferta de recomendações, estando relacionada à coordenação do cuidado entre diferentes contextos e atores da saúde. Considerar instrumentos capazes de engajar pacientes e familiares nesse processo reitera que a coprodução do cuidado engloba, além das interações interpessoais, ferramentas voltadas à orientação e à continuidade do cuidado após a alta hospitalar.

Por sua vez, a subcategoria “Continuidade do cuidado na recuperação pós-COVID-19” amplia a compreensão do fenômeno, atravessando os limites institucionais do ambiente hospitalar e ratificando que a sua sustentação depende de fatores sociais e organizacionais. Os relatos demonstram que o enfrentamento das sequelas físicas e emocionais decorrentes da COVID-19 exigiu intenso envolvimento dos participantes e de suas redes de apoio, bem como acesso oportuno a diferentes serviços e profissionais de saúde.

[...] *então, quando foi na terça-feira, eu tive alta. A enfermeira me levou ao banheiro de manhã* [...] *aí eu vi uma assadura, que acho que foi por eu não ter sido limpa direito. Quando cheguei em casa, fui tomar banho* [...] *nossa! Eu até comentei: “Gente, cheguei em casa para tomar banho, mas custei* [...] *parecia que estava tudo babando* [...] *aquela coisa toda suja e agarrada”. Me senti horrível* [...] (PD736).

[...] *sinto essa falta de ar até hoje, me atrapalha um pouco, mas, segundo o que a doutora me falou, ainda é um pouco da sequela da COVID-19* [...] *aí, nas terças e quintas-feiras, eu estou fazendo fisioterapia; inclusive, já trouxe os exercícios para casa* [...] *eu também fui a um psiquiatra* [...] *pois a noite, eu chorava muito e não conseguia dormir* (PD687).

[...] *eu precisava da marcação de um pneumologista quando já estava de alta* [...]*, e foi o que demorou um pouquinho* [...]*, eles conseguirem fazer essa marcação* [...] (PD535).

A demora no acesso a consultas especializadas e a necessidade de acompanhamento multidisciplinar elucidam que a participação ativa da pessoa no restabelecimento da própria saúde está condicionada à capacidade do sistema de saúde de oferecer suporte oportuno e responder adequadamente às demandas que surgem ao longo do processo de saúde-doença-reabilitação. Embora a coprodução do cuidado pressuponha o engajamento do paciente, sua concretização se encontra fortemente relacionada às condições que favorecem ou limitam o exercício desse protagonismo, o que acentua a interdependência entre as ações desenvolvidas no âmbito microassistencial e os elementos macroestruturais vinculados à atenção à saúde.

Juntas, as quatro subcategorias retrataram etapas distintas da jornada do paciente no cenário pandêmico e revelaram que a coprodução do cuidado nos HUFs se configurou como um fenômeno dinâmico, contínuo e relacional, construído a partir da inter-relação entre pacientes, familiares, profissionais e serviços de saúde. Outrossim, os resultados ratificam que a efetivação da coprodução abarca, simultaneamente, a valorização da experiência do paciente, o aprimoramento das práticas comunicacionais, a organização da transição entre os ambientes hospitalar e domiciliar e o compromisso do sistema de saúde em assegurar a continuidade do cuidado.

## DISCUSSÃO

O principal avanço conceitual deste estudo consiste em compreender a coprodução do cuidado como um processo longitudinal, multinível e multidimensional que articula experiências, conhecimentos e recursos distribuídos ao longo da jornada do paciente no sistema de saúde, e não apenas em momentos específicos de interação entre profissionais, pacientes e familiares. Durante a COVID-19, essa dinâmica se desenvolveu sob condições marcadas por vulnerabilidade, incertezas clínicas e reorganização dos serviços, demandando diferentes formas de comunicação, participação e compartilhamento de responsabilidades entre os atores envolvidos na atenção à saúde.

Nesse panorama, a disponibilidade e a qualidade das informações emergem como engrenagens das estratégias de coprodução do cuidado. O acesso ao conhecimento constitui um componente fundamental para a educação em saúde e para a construção de saberes em instituições e comunidades, fomentando decisões mais esclarecidas acerca da saúde e do tratamento^(20)^. Estudos apontam a necessidade de orientações sobre práticas realizadas durante a hospitalização e indicam que conteúdos claros e acessíveis podem contribuir para a redução do medo e da ansiedade, especialmente em situações de crise sanitária^(21,22)^. Além disso, a comunicação transparente entre profissionais e usuários favorece relações de confiança, promove o empoderamento e amplia as possibilidades de participação ativa na assistência^(22)^.

Todavia, a oferta de orientações compreensíveis, embora seja necessária, não é suficiente, isoladamente, para coproduzir o cuidado. Torna-se igualmente relevante considerar as percepções, vivências e preferências individuais dos usuários durante a internação hospitalar e na transição para o domicílio^(23)^. Tal perspectiva se aproxima dos pressupostos do cuidado centrado no paciente ao reconhecer sua autonomia, respeitar sua dignidade e promover seu engajamento nas decisões relacionadas ao tratamento, buscando entender suas preocupações, crenças, princípios e desejos^(24,25)^. Viabilizam-se, assim, estratégias de autogerenciamento/automanejo e a coprodução entre os distintos atores da saúde envolvidos no cuidado.

A literatura indica que a relação entre o cuidado centrado no paciente e sua experiência reflete nas práticas de coprodução, enfatizando o papel dos profissionais de saúde em avaliar o perfil de cada usuário para proporcionar um cuidado holístico e seguro, visando à excelência na jornada do paciente dentro das instituições^(26,27)^. Porém, o movimento de coprodução se concentra no estabelecimento de uma parceria de cocriação entre o prestador e o utilizador, em um sentido “micro” de maior formação de valor, considerando que a gestão do cuidado não se restringe apenas a intervenções e técnicas assistenciais internas, mas à experiência global do indivíduo no processo de saúde-doença-atenção^(27)^.

Essa compreensão reforça a relevância das orientações e instruções de alta para a transição do cuidado e para sua construção compartilhada durante a COVID-19, evidenciando o potencial de instrumentos estruturados, como planos ou cartilhas de alta, adaptados às necessidades dos usuários e às especificidades da doença^(28)^. Da mesma forma, a inclusão de pacientes e familiares nas discussões clínicas e assistenciais, em conjunto com a equipe multidisciplinar, desde a admissão até a alta, pode beneficiar o processo de tomada de decisão compartilhada e contribuir para a coprodução do cuidado e para a continuidade da assistência após a hospitalização^(25,27)^.

Em consonância com essa discussão, um estudo conduzido entre fevereiro e novembro de 2021 com 300 pacientes convalescentes da COVID-19 que receberam alta de um hospital universitário na região Sul do Brasil revelou uma avaliação positiva da transição hospital-domicílio pelos sobreviventes, embora tenham sido identificadas áreas passíveis de aprimoramento, notadamente no que concerne ao plano de alta/cuidados e aos encaminhamentos pós-hospitalização. Por outro lado, os cuidadores e as famílias manifestaram uma percepção menos favorável, atribuída às dificuldades encontradas e vivenciadas após a alta, destacando as fragilidades na transição do cuidado e a importância do envolvimento prévio nas etapas de planejamento da alta, com o objetivo de aprimorar sua execução e garantir a continuidade do cuidado em casos de COVID-19^(29)^.

Outro aspecto que merece destaque se refere às adversidades impostas pela pandemia à manutenção e à construção compartilhada do cuidado no ambiente domiciliar, ressaltando a importância do acesso aos serviços de saúde e do suporte social durante a recuperação e a reabilitação. A rápida evolução da crise sanitária, associada às frequentes mudanças nas recomendações assistenciais e às limitações organizacionais dos serviços, repercutiu na coprodução do cuidado e na continuidade da assistência prestada aos pacientes e familiares^(30)^. Paralelamente, a pandemia ressaltou e, em muitos casos, ampliou as desigualdades socioeconômicas preexistentes, influenciando o acesso aos recursos necessários ao tratamento e à recuperação da saúde^(8,9)^.

Sob essa ótica, a coprodução requer estratégias articuladas nos âmbitos assistencial, gerencial e sistêmico, capazes de considerar tanto as singularidades dos indivíduos quanto os condicionantes coletivos que permeiam a experiência do paciente no processo de saúde-doença-reabilitação, com o propósito de reconfigurar, coordenar, qualificar e assegurar a continuidade do cuidado entre os diferentes níveis de atenção^(23,28,29)^. Assim, torna-se imprescindível aprofundar o debate acerca da necessidade de modelos assistenciais e sistemas de saúde resilientes, aptos a responder a situações críticas, fortalecer os setores primário e comunitário, incorporar tecnologias inovadoras à gestão do cuidado, e integrar práticas de coprodução no contexto clínico e na gestão em enfermagem e saúde.

Levando em conta o papel da enfermagem na coordenação das ações assistenciais, este estudo sinaliza que a profissão ocupa um espaço estratégico na operacionalização da coprodução do cuidado, visto que a proximidade contínua com pacientes e familiares favorece a identificação de necessidades e dificuldades que permeiam a hospitalização, possibilitando a criação de intervenções didáticas e relacionais com foco na participação ativa dos usuários durante a assistência. Na esfera da gestão do cuidado, a discussão reforça a relevância de iniciativas institucionais voltadas ao fortalecimento das práticas de coprodução, incluindo protocolos estruturados de transição do cuidado, materiais educativos adequados à realidade dos usuários e a qualificação dos processos de comunicação.

Entre as limitações do estudo, destacam-se as entrevistas realizadas de forma remota, que podem ter influenciado a profundidade das narrativas e a compreensão de determinadas experiências relacionadas à hospitalização pela COVID-19. Adicionalmente, dificuldades técnicas ocorridas durante a gravação das entrevistas e falhas de memória relatadas por alguns participantes podem ter restringido a recuperação de determinados eventos assistenciais. Apesar disso, a estratégia adotada possibilitou o acesso a participantes geograficamente dispersos e permitiu compreender as experiências relacionadas a diferentes cenários de atenção à saúde ao longo da pandemia.

## CONCLUSÃO

Com base nos resultados, foram identificadas estratégias relevantes de coprodução do cuidado para o contexto pós-pandemia, incluindo: a promoção da educação em saúde, para que pacientes e familiares tomem decisões informadas; a valorização da experiência e da jornada do paciente, para que o cuidado seja centrado em suas necessidades; a construção compartilhada de planos de alta entre profissionais, pacientes e familiares, para que haja continuidade do cuidado; e o fortalecimento do apoio social e do acesso ao sistema de saúde ao longo do processo de saúde-doença-reabilitação. Os achados também evidenciam a importância do desenvolvimento de competências profissionais relacionadas à escuta qualificada, à tomada de decisão compartilhada e ao reconhecimento da experiência do paciente. Considerando o delineamento qualitativo e o cenário investigado, os resultados oferecem subsídios teóricos para o aprimoramento das práticas assistenciais, sem a pretensão de generalização para outros contextos.

## Data Availability

O conjunto de dados que sustenta o estudo apresentado está disponível no SciELO *Data* e pode ser acessado em: https://doi.org/10.48331/SCIELODATA.WLPY5A.

# Translation: Estrategias para la coproducción de cuidados basadas en la experiencia de pacientes hospitalizados con COVID-19: un estudio cualitativo multicêntrico*

**DOI:** 10.1590/1980-220X-REEUSP-2025-0354es

**Authors:** Jhonata de Souza Joaquim, Diovane Ghignatti da Costa, Alacoque Lorenzini Erdmann, Franciely Daiana Engel, Anderson Reis de Sousa

**Affiliations:** 1 Universidade Federal de Santa Catarina, Departamento de Enfermagem, Florianópolis, SC, Brasil.; 2 University of Ottawa, Faculty of Health Sciences, Ottawa, Canada.; 3 Universidade Federal da Bahia, Escola de Enfermagem, Salvador, BA, Brasil.

**Keywords:** Atención Dirigida al Paciente, Participación del Paciente, Estrategias de Salud, Continuidad de la Atención al Paciente, COVID-19

## Abstract

**Objetivo:**

Identificar estrategias de coproducción para la atención basadas en la experiencia del paciente durante el proceso de hospitalización por COVID-19.

**Método:**

Un estudio cualitativo, exploratorio-descriptivo, derivado de una investigación multicéntrica realizada en hospitales universitarios brasileños. Participaron treinta y cuatro pacientes dados de alta tras ser hospitalizados por COVID-19. La recopilación de datos se realizó entre mayo de 2021 y enero de 2022. Los datos se procesaron mediante análisis de contenido temático.

**Resultados:**

Se generaron 32 códigos, que dieron origen a la categoría temática “Coproducción de cuidados en el contexto de la pandemia”, compuesta por cuatro subcategorías: “Educación para la salud en la producción de cuidados”; “Coproducción de cuidados durante la hospitalización”; “Instrucciones y directrices para el alta hospitalaria por COVID-19”; y “Continuidad de los cuidados en la recuperación posterior a la COVID-19”.

**Conclusión:**

Los resultados de la investigación revelaron estrategias emergentes para la coproducción de cuidados, entre las que se incluyen la promoción de la educación sanitaria, la consideración de la experiencia y el recorrido del paciente durante la atención, el desarrollo y la implementación de un plan de alta compartido y la garantía del apoyo social y el acceso al sistema de salud en el proceso de salud-enfermedad-rehabilitación.

## INTRODUCCIÓN

La crisis global provocada por la COVID-19 no solo provocó un colapso en la salud pública, sino que también impulsó una reevaluación de los sistemas de salud, los modelos de atención y la calidad de la atención brindada^(1)^. En el ámbito hospitalario, caracterizado por hospitalizaciones prolongadas y la necesidad de cuidados intensivos durante la pandemia, la experiencia del paciente pasó a desempeñar un papel estratégico en la reorganización de los servicios de salud^(2)^.

La experiencia del paciente comprende percepciones construidas a partir de las interacciones establecidas a lo largo de la atención, reflejando incluso aspectos de la cultura organizacional de los servicios^(3)^. En este contexto, se destaca la coproducción de la atención en salud, entendida como la colaboración activa entre los actores de la salud (profesional-paciente-familia), con el intercambio de conocimientos, habilidades y decisiones en el proceso asistencial^(4,5)^.

En el ámbito hospitalario, especialmente en situaciones de mayor complejidad, como la COVID-19, la coproducción de la atención se configura como un elemento que potencia mejores resultados clínicos, al fortalecer la relación entre profesionales, pacientes y familiares, y ampliar el compromiso con la atención^(4,5)^. Sin embargo, su implementación requiere cambios en la dinámica institucional, fomentando la participación de los usuarios y el intercambio de información de manera accesible por parte de los equipos de salud^(5,6)^.

La pandemia planteó importantes desafíos para la puesta en práctica de esta coproducción, como restricciones de contacto, sobrecarga de los servicios, limitaciones en el acceso a los recursos de salud, desigualdades sociales y dificultades de comunicación, cuyos efectos se extendieron más allá del período crítico^(7,8,9)^. Dicha situación puso de manifiesto la necesidad de adaptar las estrategias de atención, a fin de garantizar su continuidad, incluso ante las adversidades.

A pesar de los avances en los debates sobre la experiencia del paciente y la coproducción de la atención, se observa una brecha en la comprensión de las estrategias que surgieron de la experiencia de los pacientes hospitalizados por COVID-19, especialmente en el contexto brasileño. Investigar estas estrategias resulta relevante para contribuir a la mejora de las prácticas asistenciales y al fortalecimiento de modelos de atención más participativos y resilientes.

Ante esto, este estudio se guió por la siguiente pregunta de investigación: ¿qué estrategias de coproducción de la atención en salud surgieron de la experiencia del paciente durante la hospitalización por COVID-19? Como objetivo, se busca identificar las estrategias de coproducción de la atención basadas en la experiencia del paciente a lo largo del proceso de hospitalización provocado por la COVID-19.

## MÉTODO

### Tipo de Estudio

Se trata de un estudio exploratorio-descriptivo, de enfoque cualitativo^(10)^, derivado del proyecto de tesis de maestría titulado «Experiencia del paciente relacionada con la atención de enfermería durante la hospitalización por COVID-19», vinculado al macroproyecto «Evaluación de la atención de enfermería a pacientes con COVID-19 en hospitales universitarios brasileños», cuyo protocolo fue aprobado por el Comité de Ética en Investigación con Seres Humanos, mediante el Dictamen n.º 4.347.463/2020 y el Certificado de Presentación para Evaluación Ética n.º 38912820.3.1001.0121.

### Lugar

El escenario de este estudio está compuesto por sectores/unidades de tres hospitales universitarios federales (HUF), ubicados en las regiones Sur y Sudeste de Brasil, en los que los profesionales de enfermería realizaron actividades de atención a personas con sospecha o diagnóstico de COVID-19. Dichas instituciones fueron elegidas por su reputación como centros de referencia en la atención de pacientes con COVID-19, y se seleccionaron de manera intencional, tomando en cuenta la afinidad entre los grupos de investigación y los investigadores, la proximidad geográfica y las diferentes realidades y experiencias en la lucha contra la COVID-19.

### Población, Criterios de Selección y Definición de la Muestra

Participaron en el estudio 34 pacientes recuperados del cuadro clínico provocado por la COVID-19, quienes recibieron alta del sector o unidad de hospitalización de los HUF. El número de participantes se definió de manera intencional, y la muestra se determinó mediante el criterio de saturación de datos^(11)^. La selección de los participantes se realizó de manera aleatoria, basándose en la lista mensual de altas de las instituciones. Se contactó por teléfono a uno de cada tres individuos, comenzando por el primero de la lista y manteniendo este procedimiento hasta alcanzar la saturación.

Los criterios de inclusión adoptados fueron: a) edad superior a 18 años; b) dominio del idioma portugués brasileño; y c) hospitalización por COVID-19 durante un mínimo de 72 horas (resultado clínico = alta hospitalaria). Los criterios de exclusión fueron: a) incapacidad para dar su consentimiento: enfermedad avanzada, uso de sedantes y alteraciones neurológicas y psiquiátricas; y b) personas debilitadas y/o angustiadas: dificultad respiratoria, dolor, posoperatorio inmediato, entre otros (condiciones verificadas en el primer contacto telefónico, con el apoyo de familiares o cuidadores)^(12)^.

### Recolección de Datos

Los datos se recolectaron de mayo de 2021 a enero de 2022 por un equipo de investigación compuesto por doctores y maestros en enfermería y en áreas de salud, además de estudiantes de pregrado y posgrado en enfermería, sin vínculo directo con los participantes. Los investigadores, previamente capacitados mediante talleres, realizaron entrevistas a distancia, por teléfono, guiadas por el «Manual para el enfoque de la entrevista a pacientes mediante la técnica del incidente crítico», con el fin de estandarizar la realización y respaldar el enfoque con información complementaria^(12)^.

El uso de la técnica del incidente crítico^(13)^ permitió identificar eventos marcantes vividos a lo largo de la hospitalización, lo que facilitó la comprensión de «situaciones», «comportamientos» y «consecuencias» (positivas o negativas) relevantes para entender la experiencia del paciente en la atención de salud. Estos elementos narrativos permitieron descripciones más detalladas de los incidentes críticos, poniendo de manifiesto las dificultades enfrentadas y revelando estrategias de coproducción de la atención a partir de las experiencias relatadas.

Las entrevistas se llevaron a cabo a partir de un guion con instrucciones para la realización a distancia, acompañado de un instrumento semiestructurado que contenía preguntas de interés relacionadas con el objeto de investigación, los datos de caracterización de los participantes, un diagrama de flujo y las orientaciones pertinentes para la entrevista^(12)^. Durante la recolección de datos, los investigadores adoptaron una postura de escucha cualificada, empática y no juzgadora, valorando las narrativas de los participantes como elementos centrales para la comprensión del fenómeno.

Se reconoce que los investigadores, debido a su formación y a sus experiencias en el ámbito de la salud, podrían influir en la producción y la interpretación de los datos. Para mitigar este efecto, se adoptó la reflexividad continua en todas las etapas del estudio, con discusiones en equipo, análisis crítico compartido y explicación explícita de los supuestos de los investigadores, con el objetivo de minimizar posibles sesgos y ampliar la fiabilidad de las interpretaciones.

Posteriormente, los datos de las entrevistas se grabaron en dispositivos de audio (duración promedio de las entrevistas = 19 minutos) y se transcribieron manualmente en su totalidad mediante el procesador de texto *Microsoft Office Word* ^®^. Se llevó a cabo un proceso de refinamiento textual, que incluyó la eliminación de palabras incomprensibles o inapropiadas. Los datos de caracterización de los participantes se registraron de inmediato al momento de la llamada telefónica, utilizando un formulario electrónico estructurado en la aplicación de gestión de encuestas *Google Forms* ^®^. A continuación, estos datos se tabularon en el programa de hojas de cálculo *Google Sheets* ^®(12)^.

### Análisis y Procesamiento de los Datos

Los datos obtenidos se analizaron mediante el análisis de contenido temático^(14)^, y la discusión de los hallazgos se basó en los marcos teóricos de la experiencia del paciente^(3)^ y la gestión de la atención^(15)^, siguiendo las siguientes etapas: a) preanálisis (organización de los datos); b) exploración del material; y c) tratamiento de los resultados (inferencia e interpretación del contenido)^(14)^. En la fase de preanálisis, se llevó a cabo la lectura y verificación de las entrevistas incluidas en este estudio, acompañada de una revisión de los fundamentos teóricos que orientaron el análisis de datos. Además, se llevó a cabo una lectura horizontal de los materiales, lo que permitió comprender la idea central de las entrevistas.

En la etapa de exploración del material, las entrevistas se incorporaron al *software NVivo 10* ^®^ (*Windows* ^®^), en el que se seleccionaron fragmentos y se crearon códigos, representados por palabras o conjuntos de palabras, para sintetizarlos. Al mismo tiempo, se elaboró una hoja de cálculo en el editor *Microsoft Excel* ^®^ para presentar el panorama general de los códigos obtenidos en el *software*, lo que facilitó la identificación de similitudes, diferencias y conexiones entre ellos.

En el procesamiento de los resultados, la inferencia y la interpretación del contenido, se generaron 32 códigos. El análisis de estos elementos, realizado mediante la herramienta de agrupamiento (*cluster*) del *software NVivo 10* ^®^(*Windows* ^®^), permitió construir un dendrograma vertical, tal como se ilustra en la Figura 1. Esta representación gráfica, estructurada en forma de árbol, permitió identificar las relaciones de proximidad y similitud entre los códigos generados.

**Figura 1:** Códigos agrupados en *cluster* por similitud de palabras – Florianópolis, Santa Catarina, Brasil, 2026.

A partir de este proceso, los códigos se reorganizaron tanto en el *software* como en la hoja de cálculo de *Microsoft Excel* ^®^, de acuerdo con la semántica de las palabras, lo que dio lugar a la categoría temática de interés y sus respectivas subcategorías. Estas, a su vez, se interpretaron a la luz de los marcos teóricos adoptados. La categorización en el *software* permitió analizar 114 fragmentos de las entrevistas, con 241 referencias asociadas a los códigos identificados. Cabe destacar que algunos fragmentos y ciertos códigos abarcaron más de una subcategoría temática, debido a la interrelación de los contenidos presentes en las entrevistas.

Con el fin de fortalecer la confiabilidad analítica de los hallazgos, se llevó a cabo el proceso analítico de manera iterativa, con lecturas sucesivas del material empírico, lo que permitió el refinamiento continuo de los códigos y las categorías emergentes, con miras a la coherencia interna y la adherencia a los datos. Con el objetivo de garantizar la credibilidad de los resultados, las categorías construidas se compararon continuamente con el material empírico, buscando asegurar su representatividad y fidelidad a las narrativas de los participantes. Por último, se llevó a cabo una revisión crítica y reflexiva de los hallazgos en el seno del equipo de investigación, lo que favoreció la profundización de las interpretaciones y la alineación teórico-metodológica.

### Aspectos Éticos

Este estudio sigue las directrices establecidas por la Circular n.º 2, del 24 de febrero de 2021, de la Comisión Nacional de Ética en Investigación^(16)^ y la Resolución n.º 466, del 12 de diciembre de 2012, del Consejo Nacional de Salud^(17)^, que regulan las investigaciones con seres humanos. Los derechos de los participantes quedaron garantizados mediante un formulario de consentimiento libre e informado, que se les leyó durante la llamada telefónica. Se garantizó la confidencialidad, y la participación fue voluntaria y sin fines de lucro. El consentimiento verbal quedó registrado en una grabación de audio. Los objetivos y métodos de la investigación se explicaron previamente. La identificación de los participantes se realizó mediante la letra P, seguida del código de ingreso hospitalario, compuesto por una letra y tres dígitos.

Cabe destacar que no se emplearon recursos de inteligencia artificial en la generación de los datos de esta investigación. Sin embargo, se resalta que se utilizó el *ChatGPT 4.0* ^®^ exclusivamente para correcciones ortográficas y gramaticales preliminares, preservando íntegramente la originalidad y las ideas del autor, debidamente referenciadas a lo largo del texto^(18)^. Además, con el fin de garantizar el rigor metodológico, este estudio sigue las directrices establecidas por la guía *Consolidated Criteria for Reporting Qualitative Research* ^(19)^.

## RESULTADOS

El estudio analizó a 34 participantes, de los cuales el 52,9 % (*n* = 18) eran hombres y el 47,1 % (*n* = 16) eran mujeres, con una edad promedio de 53 años (rango de 21 a 82 años). En cuanto al nivel educativo, el 17,6 % (*n* = 6) tenía estudios superiores, el 41,2 %, estudios de secundaria (*n* = 14), el 17,6 % (*n* = 6) tenían educación primaria (grados iniciales), el 20,6 % (*n* = 7) tenían educación primaria (grados finales) y el 3 % (*n* = 1) declararon no haber recibido educación formal o haber estudiado menos de un año. La duración de la hospitalización provocada por la COVID-19 osciló entre 3 y 90 días, con un promedio de 21 días.

A partir de la organización y clasificación de los datos, se llegó a la categoría temática «Coproducción de cuidados en el contexto de la pandemia», constituida por cuatro subcategorías (conjuntos de códigos), denominadas: (A) Educación en salud en la prestación de cuidados; (B) Coproducción de cuidados durante la hospitalización; (C) Instrucciones y orientaciones para el alta hospitalaria por COVID-19; y (D) Continuidad de la atención en la recuperación pos-COVID-19. La Figura 2, que se presenta a continuación, ofrece una representación visual de estos cuatro grupos, resaltando la interconexión entre los códigos y el alcance y la complejidad de las estrategias de coproducción de la atención identificadas en el estudio.

**Figura 2:** Contextualización del conjunto de códigos vinculados a las estrategias de coproducción del cuidado – Florianópolis, Santa Catarina, Brasil, 2026.

En el primer conjunto de códigos, “Educación en salud en la prestación de cuidados”, se pone de manifiesto que el acceso calificado a la información va más allá de la dimensión meramente comunicativa, constituyéndose como un elemento estructurante de la coproducción de los cuidados. Los relatos de los usuarios de los HUF indican que la comprensión de la condición clínica, de los procedimientos realizados y de las rutinas institucionales favorece una atención más predecible, la creación de vínculos y el desarrollo de relaciones más colaborativas entre pacientes y profesionales. Por el contrario, las situaciones marcadas por la falta de información se asociaron con la incomprensión y el distanciamiento respecto a la atención recibida.

[...] *estuve internada solo nueve días y solo recibí oxígeno. No me tuvieron que intubar. Así que ahí fue donde me recuperé más rápido, pero tengo comorbilidades y el COVID-19 es oportunista* [...] *entonces, va afectando esas enfermedades que ya tienes junto con él. Antes de contraer la COVID-19, los médicos me explicaron todo esto* [...] (PD687).

[...] *todos muy atentos* [...]*, me explicaban todo el tiempo lo que estaban haciendo. Un momento notable fue la gasometría, más por el dolor que se siente al hacérsela, pero nada fuera de lo común. Ya me sabía los procedimientos de memoria, así que para mí no hubo ninguna “sorpresa” en la atención* (PD205).

[...] *si un técnico de enfermería está conmigo* [...] *y el otro, que está con otro paciente, no está haciendo nada*[...], *aunque lo llamara, no venía a ayudarme, porque no era él quien estaba conmigo. Me parece horrible*[...] (PC1017).

Estos resultados sugieren que la información actúa como un mecanismo de redistribución del conocimiento y del poder de decisión en el contexto asistencial, creando condiciones para la participación de los pacientes a lo largo del proceso de hospitalización. Además, se observa que la educación en salud es un pilar fundamental para los demás temas identificados en este estudio, ya que la participación activa durante la hospitalización, la comprensión de las recomendaciones al momento del alta y la continuidad de la atención en el hogar dependen, en diferentes niveles, de la capacidad de las personas para asimilar y utilizar conocimientos relacionados con su propia salud.

Sin embargo, los hallazgos también demuestran que la educación en salud, aunque necesaria, no fue suficiente para garantizar la coproducción efectiva de la atención. Este aspecto queda evidenciado por la subcategoría “Coproducción de cuidados durante la hospitalización”, que revela que la participación de los usuarios está directamente relacionada con el reconocimiento de su singularidad, de sus preferencias clínicas y no clínicas, y de su capacidad para ejercer cierto grado de autonomía, lo cual es determinante para el desarrollo de prácticas de autocuidado, incluso en situaciones de enfermedad grave.

Los testimonios refuerzan la idea de que acciones aparentemente cotidianas, como decidir sobre la propia higiene o participar en el manejo del oxígeno según las indicaciones médicas, cobran relevancia al representar oportunidades concretas de negociación y cooperación en la atención. Por otro lado, surgen tensiones entre las exigencias institucionales de estandarización de las prácticas asistenciales y el deseo de los participantes de ejercer un mayor protagonismo en sus decisiones.

En circunstancias específicas, incluso cuando manifestaban interés en realizar actividades de manera independiente, prevalecían conductas profesionales más protectoras u orientadas por la lógica biomédica tradicional.

[...] *ya estaba en la sala cuando me hicieron la higiene en la cama* [...], *pero con agua fría. ¡No había agua tibia! No soporto el agua fría* [...]; *el agua tiene que estar a temperatura intermedia, aunque haga calor* [...] *entonces, me lavaba sola, era un “bañito de gato” todos los días, antes de que llegaran ellas. Me levantaba* [...] *y ya me aseaba, me cambiaba la ropa, lavaba mi ropa interior y la ponía detrás de la cama* [...] (PD687).

[...] *muchas veces, ahí con el oxígeno, uno entraba, metía el dedo y lo aumentaba* [...], *el otro venía y lo disminuía* [...], *pero la doctora ya había dicho que me dejaran ir reduciendo el oxígeno poco a poco* [...] (PA914).

[...] *me trataron muy bien. Poco a poco, me fueron ayudando y me levantaron. Lo único que puedo decir es que no me gusta ser una carga; prefiero hacer mis propias cosas. Fue entonces cuando les pedí a los enfermeros: “¿Podrían dejarme encargarme de mi propia higiene?”* [...]*; sin embargo, siguieron ayudándome* [...] (PC307).

Estas evidencias corroboran que el cuidado compartido en los HUF no se desarrolló de manera lineal, sino que estuvo marcado por negociaciones que abarcaron desde la autonomía del paciente hasta la seguridad asistencial y la organización del trabajo. Desde esta perspectiva, una hospitalización positiva puede estar relacionada con la calidad técnica de las intervenciones, con la capacidad de las instituciones para acoger e incorporar los valores, las expectativas y las preferencias de los pacientes, y con su participación en los procesos de toma de decisiones relacionados con la atención.

Cabe destacar que el “conflicto” entre la participación y el control institucional se volvió aún más explícito en los HUF en el momento del alta hospitalaria. Los elementos que componen el conjunto de códigos “Instrucciones y orientaciones para el alta hospitalaria derivadas de la COVID-19” indican que el alta representa una transición particularmente delicada para mantener la participación de los pacientes en su tratamiento. Aunque los participantes relataron sentimientos positivos asociados con el regreso a casa, los relatos revelan que la dimensión psicoemocional y cognitiva de ese momento coexiste con importantes debilidades comunicativas y organizativas.

[...] *fue muy bueno* [...] *mis doctores pasaron por la mañana y me dijeron: “¿Lista para irte?”* [...] *entonces, dije: “¡Ay, Dios mío, qué maravilla!”. Fue una alegría tan grande que el corazón me latía muy fuerte* [...] (PC1075).

*[...] regresé a casa. Entonces, él dijo [...] que debía tomar las precauciones de mantenerme aislada en la habitación* [...] *mi hija se hizo cargo de mí, ella tiene un lugar adecuado* [...] *me quedé los días necesarios* (de aislamiento) *y, gracias a Dios, no tuve ninguna complicación. La única complicación que tuve fue la caída del cabello* (PC1038).

[...] *ah, casi no decían nada. Solo mencionaban que no es fácil y que tenía que cuidarme* [...] *entonces, cuando ya me sentía mejor* [...] *le pregunté al médico: “Doctor, ¿me voy a casa pronto?”. Entonces, me fui a casa y empecé a cuidarme en casa* [...] *y luego, todo salió bien* (PA896).

La dificultad para recordar las orientaciones recibidas, la falta de materiales de apoyo y la participación limitada de los familiares en el proceso educativo revelan desafíos que trascienden la esfera individual y remiten a la forma en que los servicios articulan la planificación del alta. Así, se observa una relación directa entre esta subcategoría y los resultados relacionados con la educación en salud, ya que la efectividad de las orientaciones depende no solo de la transmisión de información, sino también de la elaboración de estrategias capaces de favorecer su comprensión e incorporación a la vida cotidiana en el hogar.

[...] *creo que deberían llamar a alguien de la familia y explicarle los cuidados, porque las personas que están en casa no tienen ni idea. Creen que la persona va a llegar a casa tal como se fue* [...] *y luego, llegas a casa y la familia “solo te recibe”* [...] *tuve un lapsus de memoria y no sabía cómo explicar ni llevar a cabo los cuidados necesarios, como, por ejemplo, la alimentación blanda, el período sin hacer ejercicio* [...] *¡Salimos de ahí y no sabemos qué hacer! El médico me dio un montón de información, pero cuando salí a la calle, ya no me acordaba de nada. Es mucha información* [...] *quizás, un folleto o un manual con algo de información para la familia* [...] *porque, por ejemplo, yo* [...] *al haber desarrollado diabetes después de una pancreatitis* [...] *”tomo” insulina y la familia necesita saber cómo aplicarla, cómo medirla* [...] (PA895).

En este contexto, la propuesta de un folleto o de un plan de alta compartido surgió como respuesta a las carencias señaladas por los usuarios de los HUF, lo que refuerza que la necesidad de este recurso va más allá de la simple oferta de recomendaciones, ya que está relacionada con la coordinación de la atención entre diferentes contextos y actores de la salud. Considerar instrumentos capaces de involucrar a los pacientes y sus familiares en este proceso reitera que la coproducción de la atención abarca, además de las interacciones interpersonales, herramientas orientadas a la orientación y a la continuidad de la atención tras el alta hospitalaria.

A su vez, la subcategoría “Continuidad de la atención en la recuperación pos-COVID-19” amplía la comprensión del fenómeno, traspasando los límites institucionales del entorno hospitalario y confirmando que su sostenimiento depende de factores sociales y organizacionales. Los relatos demuestran que hacer frente a las secuelas físicas y emocionales derivadas de la COVID-19 exigió una intensa participación de los participantes y de sus redes de apoyo, así como un acceso oportuno a diferentes servicios y profesionales de la salud.

[...] *entonces, el martes me dieron de alta. La enfermera me llevó al baño por la mañana* [...] *ahí vi una irritación en la piel, que creo que fue porque no me habían limpiado bien. Cuando llegué a casa, me fui a bañar* [...] *¡vaya! Incluso comenté: “Chicos, llegué a casa para bañarme, pero me costó* [...] *parecía que todo estaba baboso* [...] *esa cosa toda sucia y pegajosa”. Me sentí horrible* [...] (PD736).

[...] *sigo sintiendo esa dificultad para respirar hasta hoy, me molesta un poco, pero, según lo que me dijo la doctora, todavía es una secuela del COVID-19* [...] *ahí, los martes y jueves, estoy haciendo fisioterapia; de hecho, ya me traje los ejercicios a casa* [...] *también fui a ver a un psiquiatra* [...] *porque por las noches lloraba mucho y no podía dormir* (PD687).

[...] *necesitaba una cita con un neumólogo cuando ya me habían dado de alta* [...]*, y eso fue lo que tardó un poco* [...]*,que pudieran concertar esa cita* [...] (PD535).

La demora en el acceso a consultas especializadas y la necesidad de un seguimiento multidisciplinario ponen de manifiesto que la participación activa de la persona en el restablecimiento de su propia salud está condicionada a la capacidad del sistema de salud para ofrecer apoyo oportuno y responder adecuadamente a las demandas que surgen a lo largo del proceso de salud-enfermedad-rehabilitación. Aunque la coproducción de la atención supone el compromiso del paciente, su concreción está fuertemente relacionada con las condiciones que favorecen o limitan el ejercicio de ese protagonismo, lo que acentúa la interdependencia entre las acciones desarrolladas en el ámbito microasistencial y los elementos macroestructurales vinculados a la atención de la salud.

En conjunto, las cuatro subcategorías reflejaron etapas distintas del recorrido del paciente en el contexto de la pandemia y revelaron que la coproducción de la atención en los HUF se configuró como un fenómeno dinámico, continuo y relacional, construido a partir de la interrelación entre pacientes, familiares, profesionales y servicios de salud. Asimismo, los resultados confirman que la puesta en práctica de la coproducción abarca, de manera simultánea, la valorización de la experiencia del paciente, el perfeccionamiento de las prácticas comunicativas, la organización de la transición entre el entorno hospitalario y el doméstico, y el compromiso del sistema de salud para garantizar la continuidad de la atención.

## DISCUSIÓN

El principal avance conceptual de este estudio consiste en comprender la coproducción de la atención como un proceso longitudinal, multinivel y multidimensional que articula experiencias, conocimientos y recursos distribuidos a lo largo del recorrido del paciente en el sistema de salud, y no solo en momentos específicos de interacción entre profesionales, pacientes y familiares. Durante la pandemia de COVID-19, esta dinámica se desarrolló en condiciones marcadas por la vulnerabilidad, las incertidumbres clínicas y la reorganización de los servicios, lo que exigió diferentes formas de comunicación, participación y reparto de responsabilidades entre los actores involucrados en la atención de la salud.

En este panorama, la disponibilidad y la calidad de la información se revelan como elementos clave de las estrategias de coproducción de la atención. El acceso al conocimiento constituye un componente fundamental para la educación en salud y para la construcción de conocimientos en instituciones y comunidades, lo que fomenta decisiones más informadas sobre la salud y el tratamiento^(20)^. Los estudios señalan la necesidad de orientaciones sobre las prácticas que se llevan a cabo durante la hospitalización e indican que los contenidos claros y accesibles pueden contribuir a reducir el miedo y la ansiedad, especialmente en situaciones de crisis sanitaria^(21,22)^. Además, la comunicación transparente entre profesionales y usuarios favorece las relaciones de confianza, promueve el empoderamiento y amplía las posibilidades de participación activa en la atención^(22)^.

Sin embargo, ofrecer orientaciones comprensibles, aunque sea necesario, no es suficiente por sí solo para coproducir la atención. Es igualmente relevante considerar las percepciones, experiencias y preferencias individuales de los usuarios durante la hospitalización y en la transición al hogar^(23)^. Esta perspectiva se acerca a los supuestos de la atención centrada en el paciente al reconocer su autonomía, respetar su dignidad y promover su participación en las decisiones relacionadas con el tratamiento, buscando comprender sus preocupaciones, creencias, principios y deseos^(24,25)^. De esta manera, se hacen posibles las estrategias de autogestión y la coproducción entre los distintos actores de la salud involucrados en la atención.

La literatura indica que la relación entre la atención centrada en el paciente y su experiencia se refleja en las prácticas de coproducción, haciendo hincapié en el papel de los profesionales de la salud a la hora de evaluar el perfil de cada usuario para brindar una atención integral y segura, con el objetivo de alcanzar la excelencia en el recorrido del paciente dentro de las instituciones^(26,27)^. Sin embargo, el movimiento de coproducción se centra en el establecimiento de una alianza de cocreación entre el proveedor y el usuario, en un sentido “micro” de mayor generación de valor, considerando que la gestión de la atención no se limita únicamente a las intervenciones y técnicas asistenciales internas, sino a la experiencia global del individuo en el proceso de salud-enfermedad-atención^(27)^.

Esta comprensión refuerza la relevancia de las orientaciones e instrucciones de alta para la transición de la atención y para su construcción compartida durante la COVID-19, poniendo de manifiesto el potencial de instrumentos estructurados, como planes o folletos de alta, adaptados a las necesidades de los usuarios y a las especificidades de la enfermedad^(28)^.

Del mismo modo, la inclusión de los pacientes y sus familiares en las discusiones clínicas y asistenciales, junto con el equipo multidisciplinario, desde el ingreso hasta el alta, puede beneficiar el proceso de toma de decisiones compartida y contribuir a la coproducción de la atención y a la continuidad de la asistencia después de la hospitalización^(25,27)^.

En consonancia con esta discusión, un estudio realizado entre febrero y noviembre de 2021 con 300 pacientes convalecientes de COVID-19 que recibieron alta de un hospital universitario en la región sur de Brasil reveló una evaluación positiva de la transición del hospital al hogar por parte de los sobrevivientes, aunque se identificaron áreas susceptibles de mejora, especialmente en lo que respecta al plan de alta y cuidados, así como a las derivaciones posteriores a la hospitalización. Por otro lado, los cuidadores y las familias expresaron una percepción menos favorable, atribuida a las dificultades encontradas y vividas después del alta, destacando las debilidades en la transición de la atención y la importancia de la participación previa en las etapas de planificación del alta, con el objetivo de mejorar su ejecución y garantizar la continuidad de la atención en casos de COVID-19^(29)^.

Otro aspecto que merece destacarse se refiere a las adversidades impuestas por la pandemia al mantenimiento y la construcción compartida de la atención en el entorno doméstico, lo que resalta la importancia del acceso a los servicios de salud y del apoyo social durante la recuperación y la rehabilitación. La rápida evolución de la crisis sanitaria, junto con los frecuentes cambios en las recomendaciones de atención y las limitaciones organizativas de los servicios, repercutió en la coproducción de la atención y en la continuidad de la asistencia brindada a los pacientes y sus familiares^(30)^. Al mismo tiempo, la pandemia puso de relieve y, en muchos casos, agravó las desigualdades socioeconómicas preexistentes, lo que influyó en el acceso a los recursos necesarios para el tratamiento y la recuperación de la salud^(8,9)^.

Desde esta perspectiva, la coproducción requiere estrategias articuladas en los ámbitos asistencial, administrativo y sistémico, capaces de considerar tanto las singularidades de los individuos como los determinantes colectivos que impregnan la experiencia del paciente en el proceso de salud-enfermedad-rehabilitación, con el propósito de reconfigurar, coordinar, mejorar y garantizar la continuidad de la atención entre los diferentes niveles de atención^(23,28,29)^. Por lo tanto, resulta imprescindible profundizar el debate sobre la necesidad de modelos asistenciales y sistemas de salud resilientes, capaces de responder a situaciones críticas, fortalecer los sectores primario y comunitario, incorporar tecnologías innovadoras a la gestión de la atención e integrar prácticas de coproducción en el contexto clínico y en la gestión de enfermería y salud.

Teniendo en cuenta el papel de la enfermería en la coordinación de las acciones asistenciales, este estudio señala que la profesión ocupa un lugar estratégico en la puesta en práctica de la coproducción de la atención, ya que la proximidad continua con los pacientes y sus familiares favorece la identificación de las necesidades y dificultades que surgen durante la hospitalización, lo que permite crear intervenciones didácticas y relacionales centradas en la participación activa de los usuarios durante la atención. En el ámbito de la gestión de la atención, el análisis refuerza la importancia de las iniciativas institucionales orientadas a fortalecer las prácticas de coproducción, incluyendo protocolos estructurados de transición de la atención, materiales educativos adecuados a la realidad de los usuarios y la mejora de los procesos de comunicación.

Entre las limitaciones del estudio, se destacan las entrevistas realizadas de manera remota, lo que pudo haber influido en la profundidad de los relatos y en la comprensión de ciertas experiencias relacionadas con la hospitalización por COVID-19. Además, las dificultades técnicas que se presentaron durante la grabación de las entrevistas y los lapsos de memoria reportados por algunos participantes pueden haber limitado la recuperación de ciertos eventos relacionados con la atención. A pesar de ello, la estrategia adoptada permitió acceder a participantes geográficamente dispersos y comprender las experiencias relacionadas con diferentes escenarios de atención de salud a lo largo de la pandemia.

## CONCLUSIÓN

Con base en los resultados, se identificaron estrategias relevantes de coproducción de la atención para el contexto pospandémico, entre ellas: la promoción de la educación en salud, para que los pacientes y sus familiares tomen decisiones informadas; la valorización de la experiencia y el recorrido del paciente, para que la atención se centre en sus necesidades; la elaboración conjunta de planes de alta entre profesionales, pacientes y familiares, para garantizar la continuidad de la atención; y el fortalecimiento del apoyo social y del acceso al sistema de salud a lo largo del proceso de salud-enfermedad-rehabilitación. Los hallazgos también evidencian la importancia del desarrollo de competencias profesionales relacionadas con la escucha calificada, la toma de decisiones compartida y el reconocimiento de la experiencia del paciente. Teniendo en cuenta el diseño cualitativo y el contexto investigado, los resultados ofrecen fundamentos teóricos para el mejoramiento de las prácticas asistenciales, sin pretender generalizarlos a otros contextos.

## Data Availability

El conjunto de datos en el que se basa el estudio presentado está disponible en SciELO *Data* y se puede consultar en: https://doi.org/10.48331/SCIELODATA.WLPY5A.
